# Nutrition or Detoxification: Why Bats Visit Mineral Licks of the Amazonian Rainforest

**DOI:** 10.1371/journal.pone.0002011

**Published:** 2008-04-23

**Authors:** Christian C. Voigt, Krista A. Capps, Dina K. N. Dechmann, Robert H. Michener, Thomas H. Kunz

**Affiliations:** 1 Leibniz Institute for Zoo and Wildlife Research, Berlin, Germany; 2 Department of Ecology and Evolutionary Biology, Cornell University, Ithaca, New York, United States of America; 3 Smithsonian Tropical Research Institute, Balboa, Ancon, Panama; 4 Stable Isotope Laboratory, Department of Biology, Boston University, Boston, Massachusetts, United States of America; 5 Center for Ecology and Conservation Biology, Department of Biology, Boston University, Boston, Massachusetts, United States of America; The University of New South Wales, Australia

## Abstract

Many animals in the tropics of Africa, Asia and South America regularly visit so-called salt or mineral licks to consume clay or drink clay-saturated water. Whether this behavior is used to supplement diets with locally limited nutrients or to buffer the effects of toxic secondary plant compounds remains unclear. In the Amazonian rainforest, pregnant and lactating bats are frequently observed and captured at mineral licks. We measured the nitrogen isotope ratio in wing tissue of omnivorous short-tailed fruit bats, *Carollia perspicillata*, and in an obligate fruit-eating bat, *Artibeus obscurus,* captured at mineral licks and at control sites in the rainforest. *Carollia perspicillata* with a plant-dominated diet were more often captured at mineral licks than individuals with an insect-dominated diet, although insects were more mineral depleted than fruits. In contrast, nitrogen isotope ratios of *A. obscurus* did not differ between individuals captured at mineral lick versus control sites. We conclude that pregnant and lactating fruit-eating bats do not visit mineral licks principally for minerals, but instead to buffer the effects of secondary plant compounds that they ingest in large quantities during periods of high energy demand. These findings have potential implications for the role of mineral licks for mammals in general, including humans.

## Introduction

Geophagy or the consumption of soil and clay by animals is geographically widespread and known from a variety of vertebrate and invertebrate taxa including humans (summarized in [1]). Several explanations for geophagy have been postulated, including cytoprotection of the intestinal tract and preventing indigestion [2], [3], ingestion of antibiotics [4] or as an aid in mechanical digestion [5]. However, the two most important albeit possibly non-exclusive hypotheses for the consumption of fine clay involve supplementing diet with limited nutrients or buffering the effects of secondary plant compounds [6]–[14].

Many arid and tropical environments are poor in mineral contents, in part due to leaching [15]. Thus, it has often been assumed that geophagy by animals at so called salt or mineral licks serves to supplement diets with essential nutrients such as sodium, calcium or iron [2], [5], [8], [16], which may indeed explain this behavior for some species. However, species with a partial or entire diet of plant material face other challenges. Many fruits, young leaves, and other plant parts consumed by animals contain toxic, teratogenic or carcinogenic plant secondary metabolites (PSM), suggesting that frugivorous and folivorous animals may consume clay or clay-saturated water to buffer the effects of PSM [6], [11].

Bats are a special case in this context: pregnant and lactating females are under severe mineral stress [17]–[21], largely because their diets are generally low in mineral content, especially calcium [17], [22]–[24]. However, unlike offspring of most terrestrial mammals, which start consuming a partial solid diet while still suckling, juvenile bats only begin to consume a partial solid diet while still suckling, and juvenile bats only begin to consume solid food after they begin to fly and forage independently. Juvenile bats fledge only when they have almost reached adult size [25]. Thus, reproductive females often must mobilize mineral reservoirs in their skeleton as a buffer, depleting them during times of high demand and replacing them afterwards [18], [19]. For this reason, it has been suggested that some bats visit mineral licks to replace depleted mineral reserves [27]–[29].

In the Neotropics, mineral licks, also called collpas or saladeros, are small, open muddy areas that often contain running water. These licks are frequently visited by birds and mammals, including bats [27]–[29]. Frugivorous pregnant and lactating bats often visit mineral licks to take up mineral-saturated clay or water [28], [29]; therefore, reproductive female bats are assumed to compensate for low dietary mineral intake. Additionally, insects typically are more mineral depleted than fruits [23], [30]. Thus, we expected to capture more insectivorous than frugivorous bats at mineral licks. However, we found the opposite [29].

Consistent with these results is the hypothesis that frugivorous bats consume water at mineral licks to buffer the effects of PSM in their diet, as has been hypothesized for other animals (e.g. birds; [6]; elephants; [7]; humans; [14]; macaques; [11]). Due to their high food throughput, reproductive female bats, their embryos and later suckling pups might be particularly susceptible to the effects of toxic plant compounds. The diversity in diet among the neotropical bat family Phyllostomidae offered us an excellent opportunity to investigate the role of geophagy in mammals.

Omnivorous phyllostomid bats, such as *Carollia perspicillata*, feed both on insects and fruits (*e.g.* [31]), each of which could provide sources of minerals (insects [19], [20], [22], [23] and fruit [30]). *Carollia perspicillata* offers an excellent model for testing two hypotheses–geophagy for nutritional value vs. detoxification of ingested compounds–since individual *C. perspicillata* differ in the percentage of insects and fruit ingested. We hypothesized that if *C. perspicillata* visited mineral licks for the mineral enrichment of their diet, individuals feeding on a mineral poor, insect-based diet–as indicated by the relative enrichment with ^15^N in their body tissue–should be captured at mineral licks and conspecifics feeding on a more fruit-based diet should be more frequently captured at non-mineral lick (control) sites. If bats visit mineral licks primarily to consume valuable nutrients, we expected to find the opposite pattern in *C. perspicillata*. We also predicted that the obligate fruit-eating *Artibeus obscurus* [32] should have similar enrichments in ^15^N at both mineral licks and control sites. In addition, we expected to find high levels of minerals in mineral lick soils. To test this hypothesis, we compared the mineral content of clay that we collected from mineral licks with data from fruits and insects from the literature.

## Materials and Methods

We studied bats at the Tiputini Biodiversity Station in Ecuador (TBS, 0°38.31′ S, 76°8.92′ W) between 14 March and 13 April 2007. Habitat and climate are described in Voigt *et al.* [29] and Rex *et al.* [33]. We captured bats at six mineral licks and simultaneously at six arbitrarily selected control sites in the forest using ground-level mist-nets set up at a minimum distance of 50 m from the mineral licks (length 6 to 9 m; 70 dernier/2 ply, 36 mm mesh, 5 shelves, R. Vohwinkel, Velbert, Germany) between 1800 and 2100 hours. Bats were identified and reproductive status assessed as described in Voigt *et al.* [29]. All animals were released at the site of capture after collecting two small biopsies (3 mm diameter) from the wing membrane of each bat with a sterile biopsy punch (Stiefel, Germany). We never captured bats at control sites that we had previously captured at the mineral licks, and vice versa. Tissue samples were dried and stored in small plastic vials until analysis in the laboratory. Stable isotope analyses using these samples were performed at the Boston University Stable Isotope Laboratory following Voigt *et al.* [29]. Nitrogen isotope ratios of all captured species (δ^15^N) were reported in the same publication. Here we only report data for comparisons among individuals of the omnivorous *Carollia perspicillata* and the obligate fruit-eating *Artibeus obscurus*.

We collected clay and water from the same six mineral licks where bats were captured, following [34]. Mineral analyses were performed at the Cornell University Nutrient Analysis Laboratory [34]. Mineral enrichments are expressed as ppm (mg per kg) dry mass. We calculated Fisher's exact test to evaluate intraspecific differences in sex ratios between bats captured at salt licks and control sites. We tested for differences in enrichment with δ^15^N of *C. perspicillata* and *A. obscurus* captured at both types of sites with a two-tailed Mann-Whitney U-test. All work was conducted with the approval of Boston University's Animal Care and Use Committee.

## Results

**Figure 1:**
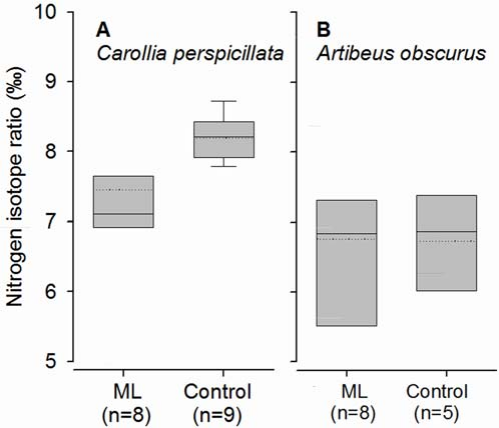
Insect content in diet is positively correlated with mineral depletion in body tissue. Nitrogen isotope ratios (δ^15^N; ‰) of the omnivorous *Carollia perspicillata* (A) and frugivorous *Artibeus obscurus* (B) captured at mineral licks (ML) and control sites (Control) at the Tiputini Biodiversity Station. *Carollia perspicillata* at mineral licks were significantly depleted in ^15^N relative to conspecifics at control sites. There was no significant difference between *A. obscurus* from mineral licks and control sites. *Carollia perspicillata* captured at control sites foraged more on insects than *C. perspicillata* captured at mineral licks or than *A. obscurus* captured at either sites. Borders of the box represent the 25 and 75 percentile, T marks the 5 and 95% percentile, solid lines within the boxes are mean values and dotted lines median values.

We captured 15 *Carollia perspicillata* (12 males/5 females) and 13 *Artibeus obscurus* (9 males/5 females). Numbers of females and males did not differ significantly between mineral licks or control sites (Fisher's exact test, *P*>0.05). The δ^15^N in wing tissue from *C. perspicillata* captured at mineral licks was significantly lower than in wing tissue of conspecifics captured at control sites (Mann-Whitney U-test: U' = 9.5, n_1_ = 9, n_2_ = 8, *P* = 0.0062; Figure 1). In contrast, the average δ^15^N of *A. obscurus* was not significantly different between bats captured at mineral licks and control sites (U' = 24, n_1_ = 8, n_2_ = 6, *P* = 0.53).

**Figure 2:**
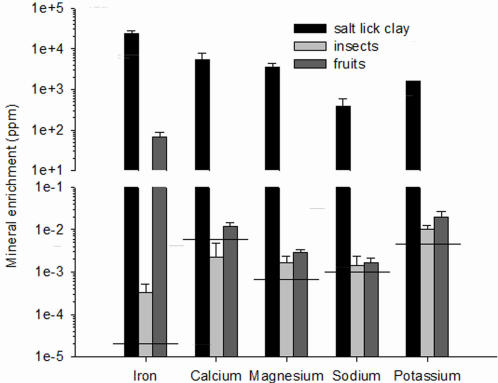
Salt lick clay contains higher concentrations of essential nutrients than insects or fruit. Content of iron, calcium, magnesium, sodium, and potassium (ppm dry mass+1 standard deviation) in clay collected from two mineral licks at the Tiputini Biodiversity Station compared to insects [23] and fruit [30] (note log scaling of y-axis). Minimum mineral requirements for growth and reproduction of small mammals are indicated by a solid horizontal line (data from National Research Council 1978 cited in [23]).

Clay at Tiputini mineral licks was enriched in five minerals (iron, calcium, magnesium, sodium and potassium) relative to fruits and insects (Figure 2). These minerals are all essential for mammalian homeostasis and reproduction. Fruits and insects were similarly enriched in sodium, potassium and magnesium, whereas calcium and iron were more enriched in fruits than in insects. Enrichment in iron varied by a factor of 10^7^, calcium and magnesium by a factor of 10^6^, and sodium and potassium by a factor of 10^5^.

## Discussion

The reasons why mammals visit mineral licks remains controversial, and could potentially vary geographically, depending on the properties of clay, as well as on the overall diet of animals. Ungulates from temperate regions, for example, show sex-specific seasonal variation in visits to licks, which appear to be associated with meeting sodium requirements during female reproduction and antler development in males [35]–[37]. High concentrations of sodium and other nutrients may also attract mammals to some mineral licks in tropical regions [7], [16,this study]. However, not all mineral licks have higher nutrient concentrations and thus the ability to buffer the effects of PSM, which may be harmful if consumed in large quantities, is an alternative hypothesis for geophagy in the tropics. Our investigation of visitation of mineral licks by two sympatric bat species with different diets, one omnivorous, one an obligate frugivore, enable us to assess the role of mineral licks for nutrition vs. detoxification in this large diverse mammalian order.

Two recent studies indicated that pregnant and lactating females of frugivorous bat species are frequently captured at mineral licks in the Amazonian rainforest [28], [29]. In both studies, it was argued that bats supplement their diet with minerals by regularly visiting licks [27], [29], because the skeleton of pregnant and lactating females is often depleted in calcium, which may limit reproduction [17], [18], [21], [38]. In small mammals, mineral requirements for growth and reproduction are 0.00002 ppm dry matter (DM) for iron in a calorically adequate diet, ca. 0.006 ppm DM for calcium, 0.0007 ppm DM for magnesium, 0.001 ppm DM for sodium, 0.0046 ppm DM for potassium (National Research Council 1978 cited in [23]). Thus, assuming a similar absorption efficiency of minerals ingested in clay, fruit or insects, all mineral requirements could be met by the three potential nutritional sources (Figure 2). Only the calcium content of insects is lower than the minimum requirements for small mammals (see also [19]. Indeed water sources with higher calcium contents are visited more frequently by reproductively active insectivorous female bats in arid regions [39], supporting this hypothesis. In this study, we demonstrate that the omnivorous *C. perspicillata* captured at control sites had higher δ^15^N than at mineral licks. Differences in ^15^N enrichment was approximately equal to one trophic level [40], [41], indicating that *C. perspicillata* encountered at mineral licks consumed mainly fruits, whereas those at control sites relied more on insects. Generally, most bats captured at mineral licks in the Amazonian lowlands are frugivorous [28], [29]. This stands in contrast to the prediction that bats with a more mineral-poor diet, namely insects, would visit mineral licks to supplement diet with nutrients such as sodium or calcium, which are scarce in rainforest environments [42].

During reproduction, female bats not only must supply their offspring with calcium and other nutrients, but they also must meet higher energy requirements [43]–[47]. Thus, female frugivorous species may ingest larger quantities of food containing PSM than non-reproductive individuals. Most bat-dispersed fruit probably contains PSM, and reproducing females may need to protect themselves and their fetus or suckling juveniles from toxic, carcinogenic, or teratogenic substances present in fruits and leaves. The buffering capacity of the clay in mineral licks clay has been demonstrated to be the most likely explanation for geophagy in birds [5], [6], primates [8], [11], elephants and other large mammals [7], [16]. However, the fact that salt licks in the Amazonian rainforest are almost exclusively visited by frugivorous species and by fruit-specialists among omnivorous species, even though their diet is more mineral-rich than that of the many animalivorous species, suggests that detoxification may be the most parsimonious explanation for bat geophagy, even though mineral supplementation may be an additional benefit of visits to mineral licks. In previous studies, no general conclusion could be drawn about the role of geophagy in the nutritional ecology of animals, including regular visits to mineral licks. Soils that comprise mineral licks vary greatly in properties such as grain size, nutrient contents, pH and, as well as the number and diversity of visiting species. The properties of mineral licks can only be directly compared if samples are collected and analyzed with a consistent method [48]. Other postulated reasons (cytoprotection, preventing indigestion, parasite and disease control, mechanical digestion aid, nutrification or detoxification) may play a role depending on location and the taxa that visit these sites. The importance of mineral licks for the well-being and reproductive success of bats and other mammals, and quite possibly also for the species richness of the landscape, is clearly great, but which species exploit mineral licks will depend on the local environment, as well as the nutritional needs and reproductive status of specific animal taxa that are present.
